# Study of the Decoherence Correction Derived from the Exact Factorization Approach for Nonadiabatic Dynamics

**DOI:** 10.1021/acs.jctc.1c00346

**Published:** 2021-06-17

**Authors:** Patricia Vindel-Zandbergen, Lea M. Ibele, Jong-Kwon Ha, Seung Kyu Min, Basile F. E. Curchod, Neepa T. Maitra

**Affiliations:** † Department of Physics, Rutgers University, Newark, New Jersey 07102, United States; ‡ Department of Chemistry, Durham University, South Road, Durham DH1 3LE, U.K.; § Department of Chemistry, Ulsan National Institute of Science and Technology (UNIST), 50 UNIST-gil, Ulsan 44919, Republic of Korea

## Abstract

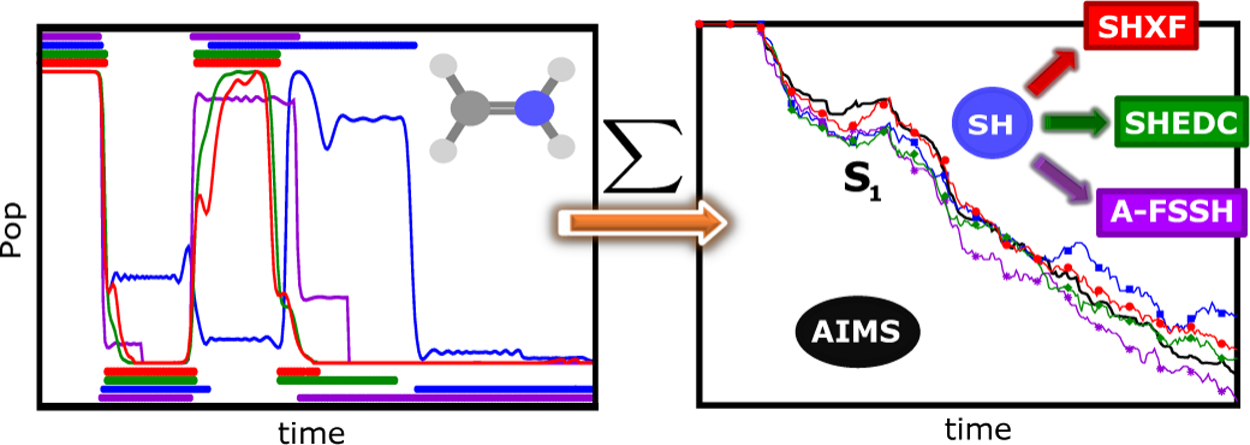

We present a detailed study of the decoherence correction to surface hopping that was recently derived from the exact factorization approach. Ab initio multiple spawning calculations that use the same initial conditions and the same electronic structure method are used as a reference for three molecules: ethylene, the methaniminium cation, and fulvene, for which nonadiabatic dynamics follows a photoexcitation. A comparison with the Granucci–Persico energy-based decoherence correction and the augmented fewest-switches surface-hopping scheme shows that the three decoherence-corrected methods operate on individual trajectories in a qualitatively different way, but the results averaged over trajectories are similar for these systems.

## Introduction

Trajectory surface hopping (SH) is one of the most widely used methods to simulate coupled electron–ion dynamics in molecules.^1−4^ While using a classical treatment of the nuclear motion, SH is nevertheless able to capture some quantum features of correlated electron–ion dynamics such as wavepacket splitting, lacking in the Ehrenfest method, another widely used classical-trajectory-based method. SH makes no a priori assumptions regarding relevant degrees of freedom and, importantly, is relatively straightforward to implement through an interface with electronic structure codes that have the capability to yield excited-state energies and gradients. At the same time, SH has an unsettling aspect in that there is a disconnect between how the electrons and nuclei evolve, a problem commonly referred to as “overcoherence”: at any given time, the nuclei evolve on a single Born–Oppenheimer (BO) potential energy surface but can instantaneously hop between them according to a stochastic algorithm dependent on the nonadiabatic coupling strengths, while the electronic evolution remains in a coherent superposition of BO states throughout. To overcome this inconsistency, several decoherence corrections have been proposed,^4−13^ which, like the SH procedure itself, are somewhat ad hoc, even if physically motivated.

The exact factorization approach,^14,15^ on the other hand, opens the possibility of *deriving* a decoherence correction from first-principles since it defines equations for a single nuclear wave function and conditional electronic wave function that exactly describe the coupled system. Reference (16) developed an SH scheme with a decoherence correction adopted from the electronic equation derived from a mixed quantum-classical treatment of the exact factorization formalism. The resulting method, SHXF, has been applied to a number of molecules demonstrating fascinating light-triggered phenomena, like for example, the photodynamics of molecular motors or the ring-opening process of cyclopropanone and cyclohexadiene.^17−20^

The performance of SHXF has not been compared yet with other decoherence corrections nor with higher-level nonadiabatic dynamics methods (aside from model systems where exact results are available^16^). Such comparisons would need some care to be meaningful. In particular, the same initial nuclear geometries and momenta should be chosen, as well as the same electronic structure method and basis set. Further, it is strongly preferable that the same electronic structure code is used since, for example, different codes utilize different convergence conditions for self-consistent field calculations that can yield quite different energies and couplings. This can be important especially when molecules evolve far from their equilibrium geometries.

In this work, we study the nature and performance of the SHXF decoherence correction on three molecules for which ab initio multiple spawning (AIMS)^21,22^ results are available. AIMS serves as a benchmark: it is based on an expansion of the nuclear wave function in terms of coupled trajectory basis functions (TBFs) (multidimensional moving frozen Gaussians), which makes it naturally free from the decoherence issue described in the SH context while yet remaining a trajectory method.^21,23−25^ This enables controlled comparisons with SH. Two of the molecules, ethylene and fulvene, represent two of the recently introduced “molecular Tully models”,^26^ while the third is the methaniminium cation. The latter is chosen because it shares the features of repeated surface crossings that the third molecular Tully model of ref (26) has (4-*N*,*N*′-dimethylaminobenzonitrile) but is easier to explore with different methods due to its smaller size. For each molecule, a comparison is made with AIMS, with the Granucci–Persico energy-based decoherence correction (SHEDC)^12,13^ and with the augmented fewest switches SH (A-FSSH)^5,27^ using precisely the same initial conditions and electronic structure methods. We find that the SHXF, SHEDC, and A-FSSH decoherence corrections operate in very different ways on an individual trajectory, but at least for the systems studied, when averaged over the full set of trajectories, the results for the electronic populations and nuclear dynamics are similar. We find that in some cases, the choice of the velocity rescaling and/or nuclear time step have an equally, if not more, important role compared to the decoherence correction. Finally, implications for further developments of mixed quantum-classical methods are discussed, but first, we begin with a brief review of the exact factorization and the SHXF method.

## SHXF

In the exact factorization approach, the full molecular wave function is represented exactly as a single correlated product, , where  are all the electronic and nuclear coordinates, respectively. The factorization is unique up to a gauge-like transformation, where the nuclear wave function χ is multiplied by an - and *t*-dependent phase, while the conditional electronic wave function  is multiplied by the inverse phase, provided the partial normalization condition  is satisfied. It can be shown that  reproduces the density and current density of the nuclear system, and we refer the reader to refs (14)(15), and (28) for more details on the formal properties of the approach, including the relation to the Born–Huang expansion.

The equations for  and  are, not surprisingly, at least as hard to solve as the full molecular TDSE;^29^ however, they offer a new starting point for approximations. One such approximation is the coupled-trajectory mixed quantum-classical (CT-MQC) approximation.^30−34^ This was derived from the exact equations in a particular gauge and taking the classical limit of the nuclear equation; this yields nuclear trajectories that satisfy classical Hamilton–Jacobi equations in a Lagrangian frame. Two further approximations are made to simplify the terms that couple the electronic and nuclear equations and are well-justified by earlier studies of the exact terms made on model systems.^31,35^ This results in a set of equations that have the form of Ehrenfest plus correction terms that depend on the nuclear quantum momentum, ∇|χ|/|χ|. Through these terms, the classical nuclear trajectories “talk” to each other and result in branching of the electronic coefficients and splitting of the nuclear wavepacket in a consistent way. Decoherence, which in a sense can be viewed as dynamics where the nuclear wavepacket motion is correctly correlated with nuclear-configuration-dependent electronic coefficients, naturally arises. CT-MQC has been demonstrated and analyzed on the one-dimensional Tully models,^30,31,34^ very recently on the photoisomerization of a retinal chromophore model,^36^ as well as on the process of ring opening in oxirane,^32,33^ where it was implemented in the CPMD code, interfaced with the density functional theory electronic structure in a plane-wave basis. Regarding computational expense, it is in a sense comparable to SH: on one hand, it is more expensive because the correction terms involve evolving trajectories and an accumulated force along any BO surface that ever gets populated, but this is compensated by needing far less trajectories to converge as it is not a stochastic method. However, while the SH approach is somehow embarrassingly parallel—each trajectory can be run fully independently—the formalism of CT-MQC imposes to run the trajectories together, requiring more computational power at the same time and effectively making it significantly slower. The quantum momentum requires input from all trajectories that are being run, that is, it is not an independent trajectory method. With further computational developments, this impediment may be able to be removed.

A second mixed quantum-classical approximation, denoted here as SHXF, was developed in ref (16), in which the electronic equation has the same form as that in CT-MQC but used within an SH framework with the nuclear trajectories evolving using forces from one BO surface at a time, instantaneously hopping between them according to the fewest-switches hopping algorithm. The correction term appearing in the electronic equation brings about decoherence in a similar way as it did in the CT-MQC algorithm but is calculated using auxiliary trajectories spawned on nonactive surfaces in order to retain an independent trajectory framework. Some details of the algorithm are presented in the following section. As mentioned earlier, SHXF has been demonstrated on a range of fascinating processes on complex molecules.^17−20^

### SHXF Equations: Decoherence and Other SH Considerations

In SH methods, an ensemble of classical nuclear trajectories are evolved, , each associated with an electronic wave function. The equation that the electronic system satisfies in SHXF is as follows:
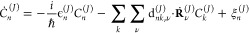
1(with terms all time-dependent), where the last term introduces decoherence, and its form differs between different schemes; for SHXF, we have
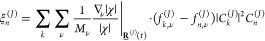
2

Above, *C*_*n*_^(*J*)^(*t*) denotes the electronic coefficient in the expansion in BO states of the electronic wave function associated with the *J*th nuclear trajectory, , while  is the BO potential energy surface evaluated at the current position of the nuclear trajectory. In the second term of eq 1,  is the nonadiabatic coupling vector (NACV) between BO states *n* and *k* with ν labeling the nucleus. The effectiveness of this coupling in causing an electronic transition is dependent on its projection along the nuclear velocity for the νth nucleus, **Ṙ**_ν_^(*J*)^. The third term ξ^(*J*)^(*t*) brings about decoherence and is given in eq 2. This depends on the quantum momentum as well as the accumulated force, that is, the difference in force along the BO surfaces integrated along the trajectory, **f**_*k*,ν_^(*J*)^ = −∫^*t*^∇_ν_ϵ_BO,*k*_^(*J*)^(*t*′)d*t*′. This term becomes effective when there is some population on more than one state, as clear from the dependence on the population factor; for example, if initially the system begins in an excitation to a single electronic excited state, the term is zero and only gets turned on after the system has evolved near a region of nonadiabatic coupling where some electronic population begins to transfer. The reader is referred to refs (31) and (34) for details on the mechanics of how this term leads to decoherence and wavepacket splitting in model systems.

Turning now to the nuclear equation, we first note that it is the same whether any decoherence correction is applied or not. For most of the time, the nuclear trajectory follows classical equations of motion along a single BO surface, the “active” surface, but instantaneously switches surfaces (“hops”) according to a prescription that depends in some way on the coupling between the states. The fraction of trajectories in the ensemble that are evolving on the *k*th surface at a given time *t*, , defines an electronic population distinct from the population obtained directly from the electronic equation, , with ρ_*kk*_^(*J*)^(*t*) = |*C*_*k*_^(*J*)^(*t*)|^2^, and in usual post-calculation analyses, it is Π_*k*_(*t*) that is ultimately recorded as the electronic population, while ρ_*kk*_(*t*) is disregarded. In the fewest-switches scheme,^1^ an expression for the hopping probability algorithm was developed by considering the requirement of “internal consistency”: that is, the average over the ensemble of many trajectories, Π_*k*_(*t*), should be equal to the average ρ_*kk*_(*t*), while minimizing the number of hops. However, since SH is run with independent trajectories, these averages are not available, and instead, the expression is applied in a stochastic sense to the individual trajectories, which breaks the internal consistency.^12^ The resulting stochastic algorithm depends on the hopping probability ζ_*ak*_ between the active state *a* and another state *k*
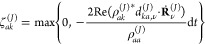
3where ρ_*ak*_^(*J*)^ = *C*_*a*_^(*J*)*^C_*k*_^(*J*)^. Then, a hop from the active state *a* to state *n* is made if ∑_*k*=1_^*n*–1^ζ_*ak*_^(*J*)^ < *r* ≤ ∑_*k*=1_^*n*^ζ_*ak*_^(*J*)^, where *r* is a random number uniformly distributed in [0, 1].

The violation of internal consistency in pure SH (i.e., eq 1 with ξ = 0) is fundamentally due to combining fully coherent electronic coefficient evolution with nuclear dynamics that in contrast evolves on a single surface at any given time, jumping surfaces stochastically. There is thus a disconnect. The nuclear trajectory in the electronic equation is the same for the coefficient associated with any surface, even though the forces as defined from the gradient of the different surfaces are different. Further, frustrated hops (see below) exacerbate the problem. Adding the decoherence correction ξ(*t*) acts to push the electronic coefficients to the active state, dampening them on the nonactive surfaces. As mentioned before, the SHXF correction can be derived from the exact factorization equations.

We briefly discuss some key aspects of how the SHXF correction is computed; full details can be found in ref (16). To retain an independent trajectory description, auxiliary trajectories are used to evaluate the quantum momentum appearing in the decoherence term in the SHXF equation.^16^ For each independent trajectory, an auxiliary trajectory is generated on the nonactive surfaces when the population of that surface becomes nonzero (or above a small threshold). The auxiliary trajectory is launched with a velocity such that the difference in potential energy from the active surface is isotropically distributed in the coordinates, and this velocity then steps forward the position of the auxiliary trajectory. In this way, the calculation of gradients of auxiliary surfaces is avoided, aiding in computational efficiency. In a similar spirit, the accumulated force along a surface is calculated from directly computing the change in momentum over a time step. The quantum momentum is obtained by considering a Gaussian of isotropic width σ centered at each auxiliary trajectory, from which follows that the quantum momentum is given by the distance of the average of the auxiliary trajectory positions, weighted by the populations, to the actual trajectory’s position.

There is clearly a significant numerical cost reduction in using auxiliary trajectories to compute the quantum momentum instead of actually coupling the different surface hopping trajectories. A price to pay for this is the introduction of the parameter σ. We avoid empiricism by fixing it to be the width of the ground-state nuclear wavepacket at the initial equilibrium geometry.

#### Other Decoherence Schemes

We will compare the effect of the SHXF ξ(*t*) on the dynamics to two widely used decoherence corrections, SHEDC and A-FSSH, which we now briefly discuss.

The SHEDC decoherence correction has quite a different form from SHXF, acting directly on nonactive states to damp the amplitude on them at a rate that depends on the energy gap  between the surfaces and the kinetic energy *T* of the nuclei.^10,12,13,37^ It is imposed as an exponential decay of amplitudes on the nonactive state, which, if written in the form of eq 2, would correspond to an effective
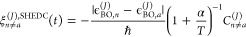
4while for the active state *a*, the coefficient is adjusted so that the sum of all coefficients is 1. The parameter α is a constant and could be adjusted but mostly is fixed as 0.1 H.^10^ It should be noted that although the original papers proposed to apply this decay to the populations, in some versions of widely used codes, such as the one we use here, the correction is applied to the coefficients. However, numerical comparisons between the two approaches for a subset of molecules do not reveal significant practical differences in the results.^26^

In another contrasting approach, A-FSSH defines a decoherence rate based on considering how fast trajectories evolving on different surfaces move away from each other;^27^ this was motivated by a comparison with the quantum-classical Liouville equation.^5^ Each trajectory carries with it auxiliary trajectories evolving on different surfaces, which are propagated classically, similar to SHXF. In A-FSSH, however, the electronic coefficient is collapsed to a state in a stochastic manner, as determined by a decoherence rate computed from
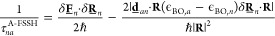
5where  is the position of the trajectory on auxiliary surface *n* relative to the position of the trajectory on the active surface *a*,  is the difference in BO forces on surface *n* and *a*, and everywhere in the equation, the dot product implies, for example,  and .

If we were to write this as an effective decoherence term in eq 2, we would have ξ_*n*≠*a*_^(*J*),A-FSSH^ = −*C*_*n*_^(*J*)^/τ_*n*≠*a*_^A-FSSH^. However, the rate is instead used in a stochastic procedure: if d*t*_c_/τ_*n*≠*a*_^A-FSSH^ is larger than a random number, then the amplitude *C*_*n*_ is collapsed to 0 on state *n*, while that on the active state is increased so that the sum of the coefficients remains 1. A separate reset rate is used to then reset  to 0.

The three decoherence corrections, exact-factorization-derived SHXF, the energy-based SHEDC, and the stochastic coefficient collapse of A-FSSH, could not appear more different! Indeed, we will find in that in practice, the way that the three decoherence corrections above act on the trajectories is very different. Still, after averaging over the SH trajectories, the populations and geometries (not shown here) are similar for the systems studied here.

We next turn to some other issues that any SH algorithm, decoherence-corrected or not, must confront.

#### Convergence Questions

The stochastic hopping process implies that several trajectories for each initial condition should be run, and convergence to a given standard error has to be monitored carefully. It requires typically tens to hundreds of trajectories per degree of freedom.^1,38^ Further, there is the question of the time step required for convergence: the hopping probability at a given time step clearly decreases linearly as the nuclear time step d*t* decreases; however, the system is interrogated whether it wants to hop correspondingly more often so that it is believed that these two effects compensate. However, for very localized avoided crossings or conical intersections, the hopping can be missed unless d*t* is taken too small to be practical; how many electronic time steps are used within this d*t* is also an important factor, including how the interpolation for the electronic propagation is done within d*t*. Using a wave function overlap-based approach with local diabatization to obtain the couplings can improve the numerical stability.^39−42^ Reference (43) very recently showed that the stochastic algorithm tends to overestimate the hopping rate when the hopping probability is large, and instead, a modified scheme based on a cumulative hopping probability rather than the instantaneous one was proposed that significantly reduces the sensitivity to the time step, as well as requiring less trajectories for convergence.

#### Momentum Adjustment

The SH algorithm in itself lacks a firm first-principles derivation (although see ref (44) for recent progress), and as a consequence, there are aspects of the nuclear dynamics which need to be adjusted in some way. One important aspect is the velocity adjustment after a hop. It is asserted that each trajectory should satisfy energy conservation, where the gain or loss in the potential energy is compensated by a loss or gain in the kinetic energy, but there is no unique way to achieve this.^45−47^ Two common ways are isotropic rescaling and rescaling along the NACVs between the two states **d**_*an*_. We note here that in other trajectory-based schemes where the trajectories are coupled rather than independent, such as in AIMS or CT-MQC, energy conservation of an individual trajectory would not be required. In AIMS, the nuclear velocities of a newly spawned TBF are scaled per default along the NACV. AIMS was shown to be insensitive to the rescaling process—isotropic rescaling produces similar results to the NACV one.^26^

In isotropic rescaling, every velocity after the hop is scaled uniformly such that the total energy is conserved: with ν labeling the atom, **Ṙ**_ν_ → κ**Ṙ**_ν_, where  and the trajectory hops from surface *k* to surface *n*. Rescaling along the NACV is believed to be theoretically more justified from semiclassical arguments.^48−50^ In this case, **Ṙ**_ν_ → **Ṙ**_ν_ + γ**d**_ν,*kn*_/*M*_ν_ where γ is determined by the quadratic equation resulting from equating the sum of the nuclear kinetic and potential energies on surface *k* to that on surface *n*.

If the potential energy gain after the hop exceeds the kinetic energy, then the hop is rejected. In this case, some works argue that the nuclear momentum should then be reversed, but other works argue that it should be kept as is.^38,39,45,51^ There are generally more rejected (a.k.a. frustrated) hops when rescaling along the NACV is done since only the kinetic energy along the NACV is available, and this can result in a poorer internal consistency; moreover, the NACV is not always accessible from the electronic structure code being used. On the other hand, a disadvantage of isotropic scaling is that it is size-extensive: even if the dynamics involves just a few atoms of a large molecule or cluster, the rescaled velocity unphysically depends on the entire kinetic energy even of atoms that are not involved in the process. These factors suggest a third rescaling procedure: scale via NACV, and when the hop is forbidden, then apply isotropic scaling. We refer to this as “NACV + iso” in the following sections (in fact, the rescaling option denoted as “NACV” in the Newton-X code does NACV + iso, while the corresponding option in SHARC, which we use in this work, does NACV).

It is well-worth noting that there is an SH scheme with interacting trajectories, consensus surface hopping,^52^ where the hopping probabilities are determined by collective input from the entire ensemble of trajectories, which avoids the somewhat ad hoc momentum adjustment needed in usual SH as well as not needing decoherence corrections. An approximate version of this, quantum trajectory surface hopping,^53^ uses independent trajectories, while still avoiding momentum rescaling.

## Computational Details

With SH and SHXF, calculations are performed with the code PyUNIxMD (UNIversal eXcited state Molecular Dynamics).^54^ The current capabilities include BO, Ehrenfest, SH, and SHXF dynamics, interfaced with a range of electronic structure programs. Since the main objective of the present work is to compare the effect of the decoherence correction derived from exact factorization with SHEDC, A-FSSH, and against the high-level AIMS method which we consider in this work as a reference, we keep other aspects of the calculations the same as much as possible. In particular, for the electronic structure, we use CASSCF implemented in MOLPRO^55^ for our calculations on ethylene (SA(3)-CASSCF(2/2)), the methaniminium cation (SA(2)-CASSCF(6/5)), and fulvene (SA(2)-CASSCF(6/6)) with the 6-31G* basis set. The SHEDC and A-FSSH computations are done with the code SHARC 2.0 (surface hopping including arbitrary couplings).^56−58^

The initial conditions for the nuclear coordinates and velocities are taken exactly the same as in the AIMS calculations,^26^ which is Wigner-sampled from uncoupled harmonic oscillators of frequencies determined from the optimized ground-state geometry of the molecule. For ethylene and the methaniminium cation, both geometries and momenta were sampled from this distribution, while for fulvene, just the geometries were Wigner-sampled and initial momenta were set to 0. Every trajectory was averaged using different random seeds to enable the convergence of the FSSH stochastic process; the total number of trajectories for each molecule is detailed below.

The nuclear time step is taken as d*t* = 0.5 fs unless otherwise stated. We have checked that decreasing the time step does not alter the results except for the case of fulvene; the convergence is generally better for the decoherence-corrected schemes than for uncorrected. As will be discussed, the dynamics in fulvene is somewhat sensitive to the choice of time step. The large slope of the crossing region implies that a large number of trajectories encounter the sharp and localized nonadiabatic coupling.

For SH and SHXF, the explicit NACVs were used in the equation of motion, while for A-FSSH and SHEDC, they were obtained from wave function overlaps by default in SHARC.^59^ We checked that there is little difference in the results when using these two approaches, except for the fulvene molecule where the convergence with respect to time step is better using the wave function overlap scheme, as mentioned earlier. An isotropic velocity adjustment was performed after a surface hop unless otherwise stated.

The population traces for AIMS were taken from ref (26) for ethylene and fulvene. For the methaniminium cation, AIMS dynamics was performed with the MOLPRO/FMS90 interface^60^ using an adaptive time step of 20 a.u. (5 a.u. in regions of nonadiabatic coupling) and an SA(2)-CASSCF(6/5)/6-31G* level of theory for the electronic structure (mirroring the electronic structure used for the mixed quantum/classical methods). The AIMS parent TBFs were started from the same set of initial conditions as the other nonadiabatic methods.

## Results

Our main objective is to compare the effects of the decoherence correction arising from the exact factorization to the widely used SHEDC and A-FSSH.

### Ethylene

As discussed in ref (26), dynamics after photoexcitation to the S_1_ state represents a molecular Tully-1 system since it proceeds through a single nonadiabatic event through a conical intersection. This represents a cis–trans-like isomerization of the molecule through a twisted and pyrimidalized geometry.^21,61^ The importance of having consistent initial conditions and electronic structure methods in comparing different dynamics methods for this molecule are emphasized in ref (26), and here, we use the same 66 initial conditions, geometries, and momenta used there from the Wigner-sampled ground-state geometry. We ran 10 trajectories for each initial condition, but note that the results were essentially converged even with 5 trajectories per initial condition. The width of the Gaussian, σ, is obtained from the initial distribution of the nuclear trajectories of the CC double bond and set to 0.05 a.u.

**Figure 1:**
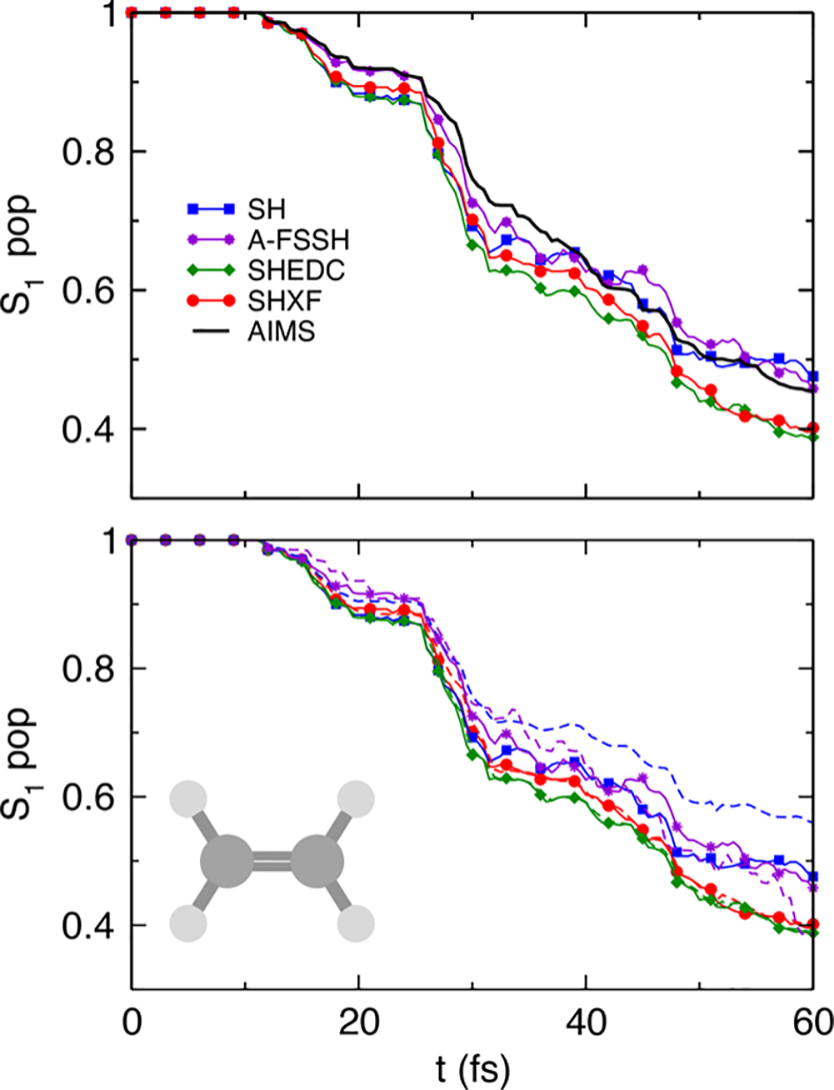
Population dynamics in ethylene: SHXF compared with SH, SHEDC, and A-FSSH, all with isotropic velocity adjustment, along with the reference AIMS results (from ref (26)). The top panel shows the fraction of trajectories Π_S_1__(*t*) in the S_1_ state. The lower panel demonstrates the internal consistency of the SH methods, with the solid lines showing Π_S_1__(*t*) again, compared with dashed lines showing the S_1_ electronic populations ρ_S_1_,S_1__(*t*).

In Figure 1, we plot the S_1_ populations as determined by both the fraction of trajectories and the electronic populations, computed from the SH, SHXF, SHEDC, and A-FSSH simulations. For this system, the fraction of trajectories predicted by uncorrected SH is very close to the reference AIMS, but we see that there is a notable internal consistency error, as expected. Averaged over trajectories, the SHXF decoherence correction from exact factorization and SHEDC yield very similar results, increasing the population transfer compared to the uncorrected SH and correcting the internal consistency of the uncorrected SH (the electronic populations are practically on top of the fraction of trajectories in both cases). They appear to agree less well with AIMS but do not deviate too far and would lie within the standard error of AIMS.^26^ A-FSSH is closer to AIMS, but it shows worse internal consistency than SHEDC and SHXF.

**Figure 2:**
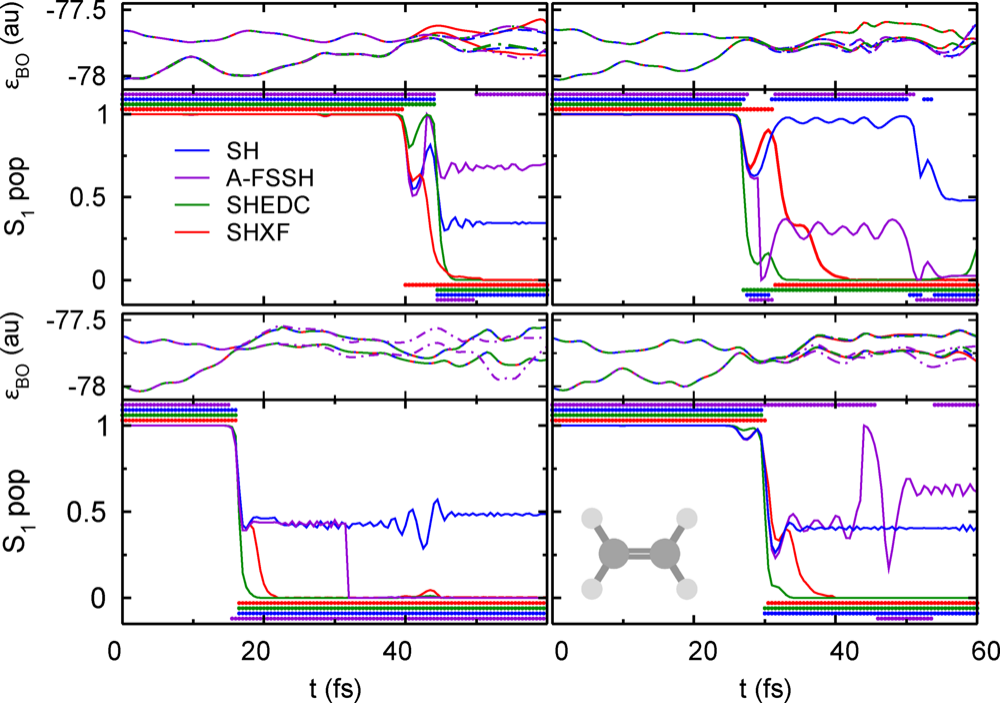
Comparing population dynamics in ethylene for four trajectories with the same initial conditions, SH, SHXF, SHEDC, and A-FSSH, with isotropic velocity adjustment. Continuous lines show the populations ρ_S_1_,S_1__(*t*), while the correspondingly colored symbols indicate the active state. Top panels show the electronic energies during SHXF dynamics. The appendix gives an AIMS analogue for this.

The close agreement of SHXF, SHEDC, and A-FSSH is not obvious, given the different structure of the corrections discussed earlier. Indeed, on an individual trajectory level, their behavior is quite different. In Figure 2, we show the populations and active state for four randomly chosen trajectories in the SH, SHXF, SHEDC, and A-FSSH simulations. The SHEDC correction damps down the populations after a hop in a mostly (but not entirely) monotonic way, while SHXF tends to be typically nonmonotonic, showing more oscillations, and generally takes longer to decohere. The stochastic nature of the A-FSSH decoherence correction is clearly evident in the plots and suggests, for this molecule, a longer decoherence time than the other methods. The appendix provides an analogue to this figure for the AIMS calculations, including a discussion highlighting essential differences between SH methods and the AIMS approach.

**Figure 3:**
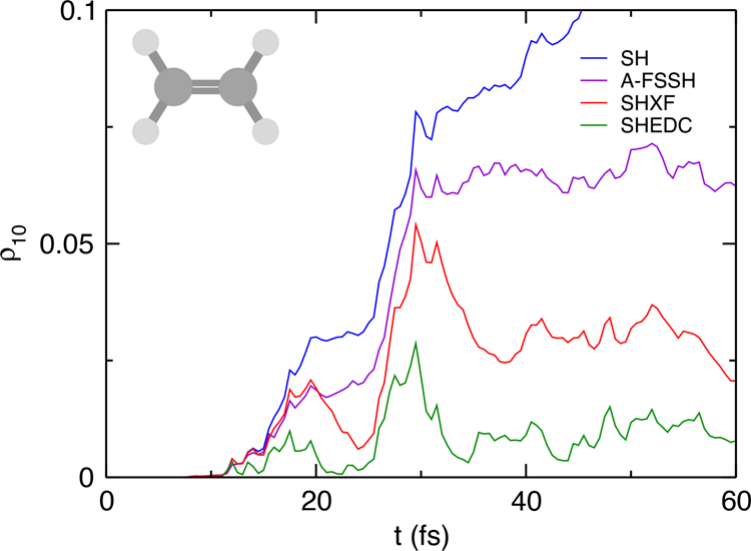
Decoherence indicator in ethylene: SH, SHXF, SHEDC, and A-FSSH.

The different behavior on an individual trajectory level is reflected in an average over all trajectories of the decoherence indicator,^30−32^ defined as  (see Figure 3). The SHXF dynamics grows to a larger coherence and takes a longer time to decohere than SHEDC, but the overall structure is similar. The coherence peak around 17 fs reflects a small number of trajectories that reach a conical intersection earlier than those associated with the second peak around 30 fs. On the other hand, as clear from the sample trajectories, A-FSSH remains coherent longer. Although in the present case, this difference does not affect the overall population dynamics very much nor the nuclear geometries (not shown), it opens the question of whether the different behavior results in other systems.

**Figure 4:**
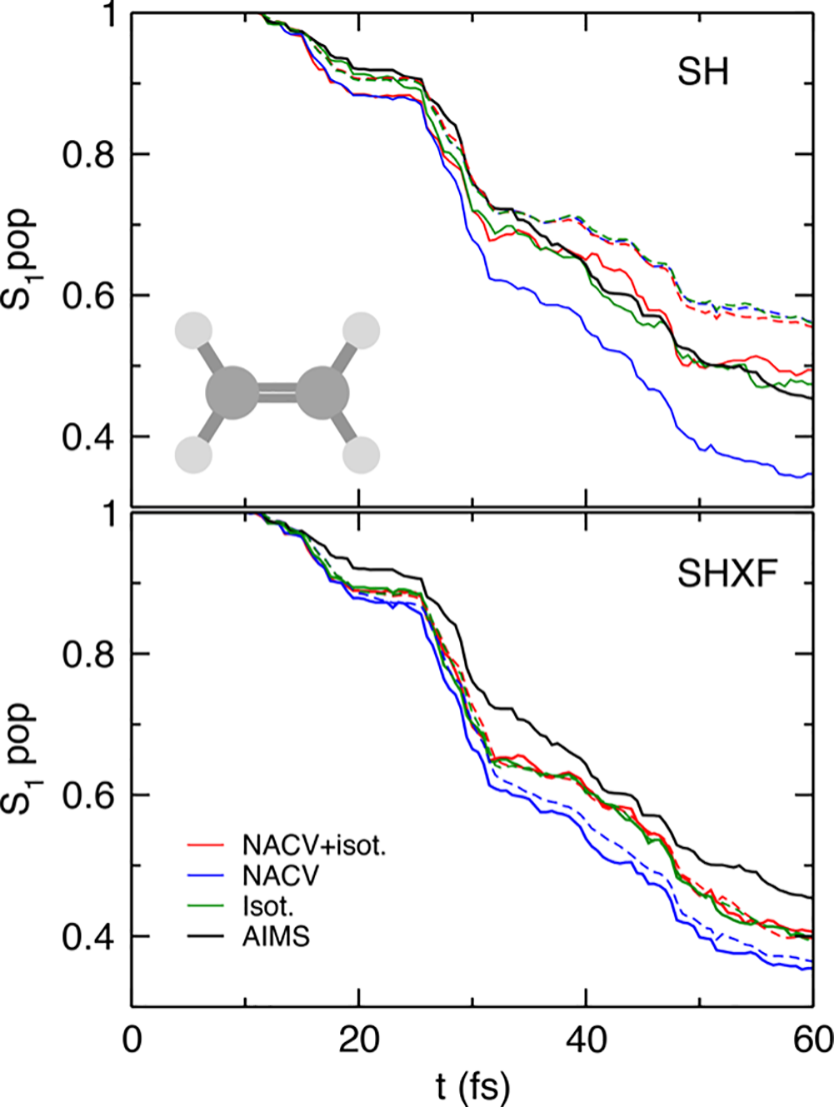
Comparison of different velocity adjustments in ethylene. Top panel: uncorrected SH, Π_S_1__(*t*) and ρ_S_1_,S_1__(*t*), with velocity adjustments of isotropic, NACV, and NACV-iso; lower panel: the same with SHXF. AIMS is shown as a reference.

Finally, the importance of the choice of velocity adjustment is evident in Figure 4, where the top panel shows the results of uncorrected SH with three different ways of velocity adjustment and the lower panel shows the SHXF case. The spread in the results shows that in this case, the choice of velocity adjustment has just about as much effect on the dynamics as the decoherence correction. In particular, while the internal consistency is very well corrected by the decoherence correction when using isotropic scaling, errors remain when scaling along NACV is performed, consistent with the expectation from the earlier discussion on velocity adjustment. When isotropic scaling is used as a “back-up” to scaling along the NACV in the NACV + iso approach, the error in the internal consistency is again small when the decoherence correction is applied; the results are close to the isotropic scaling case for this molecule.

### Methaniminium Cation

Despite its apparent similarity to ethylene (isoelectronic and planar but here with a CN double bond), the dynamics of the methaniminium cation after photoexcitation to S_1_ is quite different: following initiation of the photoisomerization after the excitation, the methaniminium cation typically meets another region of nonadiabatic coupling in a different region of configuration space, displaying recrossings with S_1_ before decaying to S_0_.^62^ The molecule tends to show torsional motion, and the initial transfer of population to S_0_ occurs once the system rotates around the CN bond from 0 to 90° (this contrasts with the photodynamics obtained by exciting the molecule to the S_2_ electronic state, where bond elongation couples with rotation^62^). Here, we use 70 initial conditions, each repeated 4 times. Preliminary trajectory runs indicate that a time step of 0.25 fs leads to converged results with respect to time step. The parameter σ is set to 0.056 a.u., which is the uniform variance obtained from the initial distribution of the CN bond of the nuclear trajectories.

**Figure 5:**
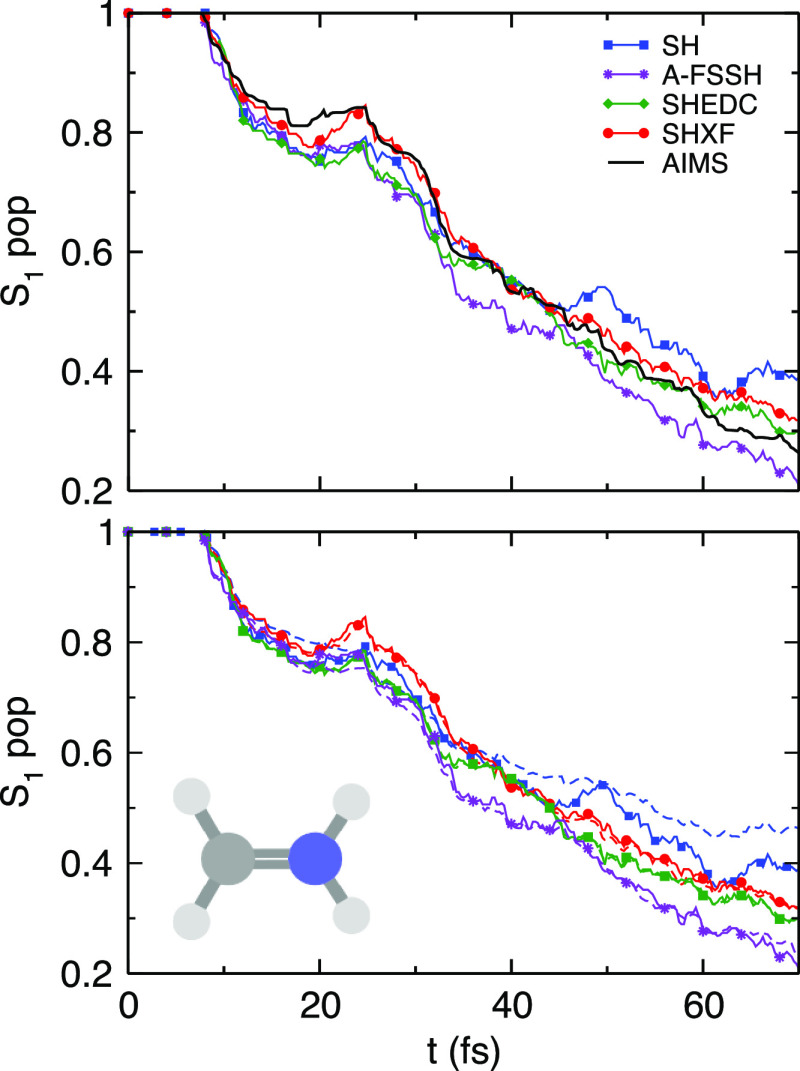
Population dynamics in the methaniminium cation: SHXF compared with SH, SHEDC, and A-FSSH, all with isotropic velocity adjustment, along with the reference AIMS result. The top panel shows the fraction of trajectories Π_S_1__(*t*) in the S_1_ state. The lower panel demonstrates the internal consistency of the SH methods, with the solid lines showing Π_S_1__(*t*) again, compared with dashed lines showing the S_1_ electronic populations ρ_S_1_,S_1__(*t*).

Figure 5 shows the population dynamics in SHXF as compared with SH, SHEDC, and A-FSSH, all using isotropic velocity adjustment, along with the reference AIMS. After some fast transfer around 10 fs, where the molecule initiates a direct photoisomerization to S_0_, the populations then plateau with recrossings back to S_1_ before then steadily transferring to S_0_, as mentioned earlier.

The poor internal consistency of the uncorrected SH is evident after the first transfer and especially at later times. The overcoherence of uncorrected SH impacts the populations at later times, yielding less transfer to S_0_ than AIMS and the decoherence-corrected SH methods. The decoherence-corrected methods correct this, with SHXF in particular giving the best overall agreement with AIMS. The SH methods all transfer at a similar but slightly greater rate than AIMS initially, and then, SHXF matches AIMS very closely after a greater S_0_ → S_1_ population transfer around 25 fs in the second interaction region, while SHEDC and A-FSSH hesitate in their steady transfer to S_0_. At longer times, A-FSSH overshoots the population transfer.

**Figure 6:**
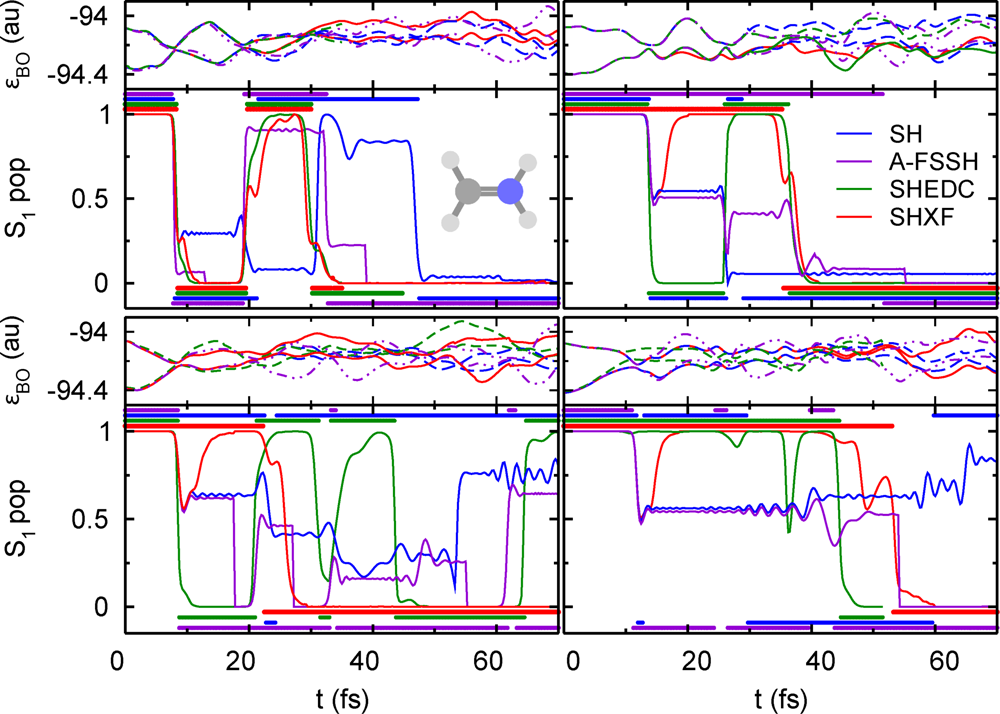
Comparing population dynamics in the methaniminium cation for four trajectories under the same initial conditions, SH, SHXF, SHEDC, and A-FSSH, with isotropic velocity adjustment. Continuous lines show the population ρ_S_1_,S_1__(*t*), while the correspondingly colored symbols indicate the active state. Top panels show the electronic energies during SHXF dynamics.

**Figure 7:**
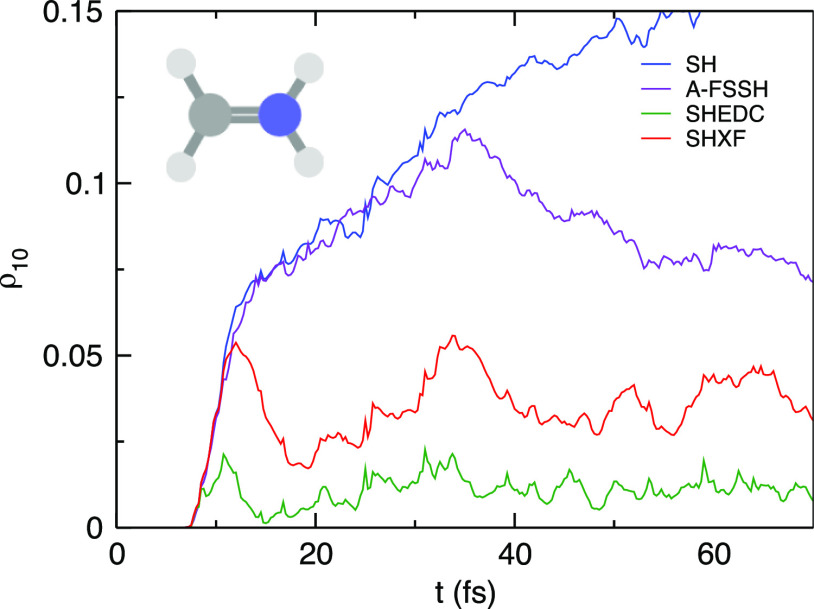
Decoherence indicator in the methaniminium cation: SH, SHXF, SHEDC, and A-FSSH.

Again on an individual trajectory level, the decoherence corrections act in different ways on the electronic populations, as evident from the sample of trajectories shown in Figure 6, and this is again reflected in the trajectory-averaged quantity, the decoherence indicator, shown in Figure 7. Again, SHXF shows a similar coherence structure to SHEDC but reaches larger values, while A-FSSH is somewhat different and takes longer to decohere. Figure 6 also highlights further how the recrossings between S_0_ and S_1_ states lead to a more severe deviation of SH from internal consistency (Figure 5) than for ethylene, affecting the population transfer as noted earlier.

### Fulvene

Fulvene represents a challenging case: after photoexcitation to the S_1_ state, there are two possible pathways for an ultrafast internal conversion to the ground state.^26,63,64^ One involves a peaked conical intersection reached by a twist of the C=CH_2_ bond, while the other involves a strongly sloped conical intersection reached by the stretch of the C=CH_2_ bond.^26^ The latter results in a transfer to S_0_ and subsequent reflection back toward the same nonadiabatic region and population transfer back to the S_1_ state. This second pathway resembles the Tully model III, and as in ref (26), we choose the initial conditions to favor this. The σ parameter is chosen as 0.065 a.u., which corresponds to the variance of initial distribution of CC double bonds of the nuclear trajectories.

**Figure 8:**
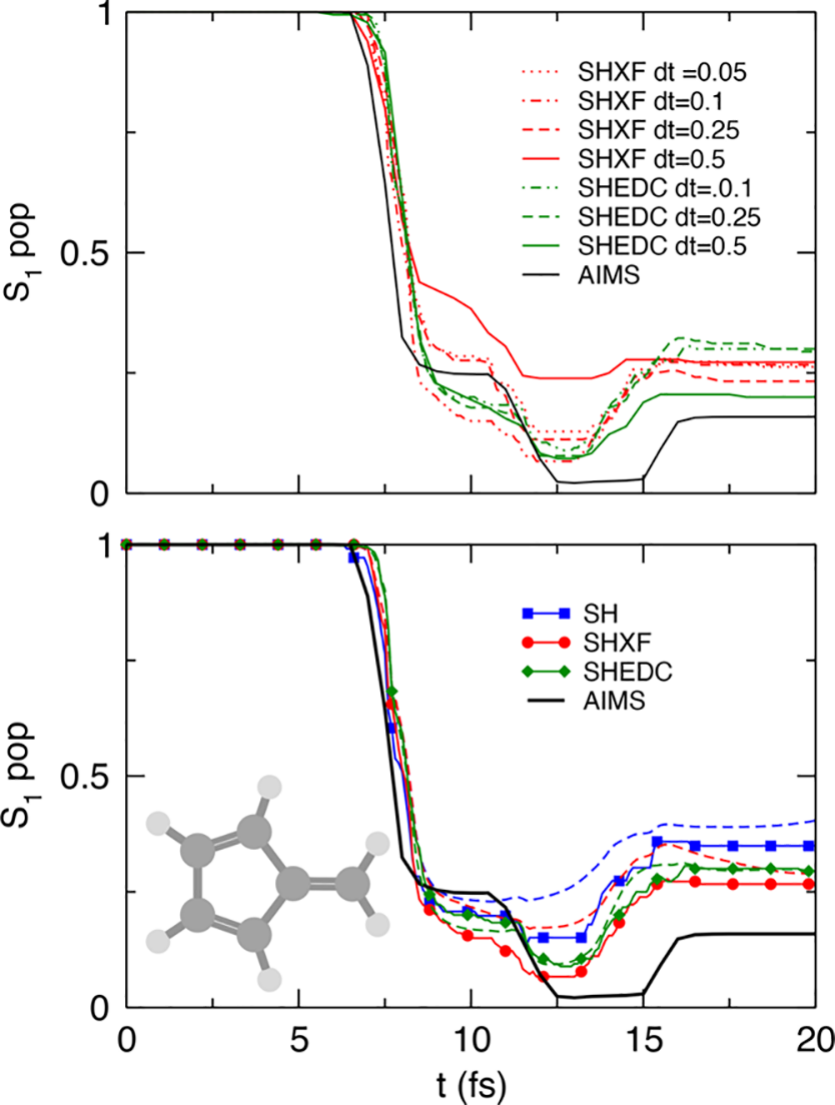
Fulvene populations. The top panel shows the convergence of Π_S_1__ with respect to the nuclear time step d*t* = 0.05, 0.1, 0.25, 0.5. Lower panel: choosing d*t* = 0.1, we plot the fraction of trajectories Π_S_1__ along with ρ_S_1_,S_1__ (dashed) for SHXF, SHEDC, and SH against the AIMS reference.

The sharply sloped conical intersection gives a large dependence on the time step d*t* since the interaction region can be missed. We see that as d*t* decreases from 0.5 to 0.25 to 0.1 fs, SHXF predicts more population during the initial event (Figure 8, top panel) but that the d*t* = 0.05 fs result is closer to the d*t* = 0.25 fs result than to the d*t* = 0.1 fs result; the results are thus not fully converged with respect to the time step. To some degree, this dependence can be mitigated by using wave function overlaps to compute the coupling terms, with a local diabatization scheme. The SHEDC calculations in SHARC utilize this scheme, and we see in the top figure that although SHEDC predictions with d*t* = 0.5 fs (green dash-dot line) plateau to a different level after 15 fs (and is closer to the AIMS result) from that predicted with the d*t* = 0.1 and 0.25 fs calculations, the results do appear converged with d*t* = 0.25. This example highlights the need to check for convergence with respect to the time step in these cases. As mentioned earlier, the recent method of ref (43) is promising in this regard. We note that AIMS uses an adaptive time step and so does not have such sensitivity.

In the lower panel, we see that both decoherence-corrected schemes increase the population transfer compared to pure SH, with good internal consistency. Both SHEDC and SHXF agree quite well with each other, despite their different operation mechanisms.

Finally, it was observed in ref (26) that the dynamics heavily depends on the choice of velocity adjustment. Isotropic scaling gives results notably worse than scaling along the NACV for this molecule, which might be explained due to the larger size of the molecule, since the problem with unphysical redistribution of the kinetic energy in the isotropic method becomes more important. The results shown in Figure 8 used scaling along the NACV.

## Conclusions

Overall, the results show that SHXF provides a useful improvement over uncorrected SH in comparison with the reference AIMS and that it gives a similar behavior for observables as SHEDC and A-FSSH. We have found that the three decoherence corrections suggest strikingly different mechanisms on an individual trajectory level. This was clear in both the form of the corrections and their demonstrated behavior on the molecular systems. For the systems studied, although there are some small differences when more than one interaction region is encountered, the different decoherence mechanisms nevertheless on the whole yielded similar population dynamics once averaged. This appears unlikely to be true generically, given their different modes of operation. Whether one can somehow predict when the differences will lead to significantly different observables and why they were similar here is a question for future research.

Several ad hoc aspects of the SH approach itself, arising from the fact that SH itself is not an algorithm derived consistently from first-principles, make it difficult to give a definitive and unambiguous assessment of the corrections themselves, and in some cases, issues such as velocity scaling procedures, for which different procedures have been argued to be the best, give larger differences than the decoherence corrections themselves; indeed in some cases, SH without decoherence performs similarly. Similar observations have been independently made in two very recent papers studying the traditional decoherence methods.^47,65^ Further, the SH scheme is unable to correctly describe situations where several surfaces are parallel while others are not such that the (de)coherence should be considered in a state-pairwise scheme rather than as an overall correction per state.^66^ Thus, in parallel to further exploring SHXF and its capabilities, especially for large systems given its computational efficiency, further developments of CT-MQC and alternative practical mixed quantum classical methods from the exact factorization are an avenue for future work.

## Appendix: Analysis of AIMS Runs for Ethylene

We present here an AIMS analogue of Figure 2 for ethylene. In AIMS, the nuclear wave function for each BO state is described by a linear combination of frozen Gaussians, the so-called TBFs
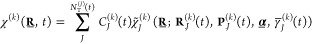
6where  are multidimensional Gaussians, each associated with a time-dependent complex coefficient *C*_*J*_^(*k*)^(*t*), where *J* labels a specific TBF, evolving in electronic state *k*. The phase-space center of each multidimensional Gaussian function is given by  and momentum . The matrix  contains the widths (the same for all TBFs and independent of the electronic state), and γ̅_*J*_^(*k*)^(*t*) is a phase. The TBFs evolve along classical trajectories, and the spawning algorithm will increase the size of the TBF basis when nonadiabatic regions are encountered (see ref (23) for additional details on AIMS).

**Figure 9:**
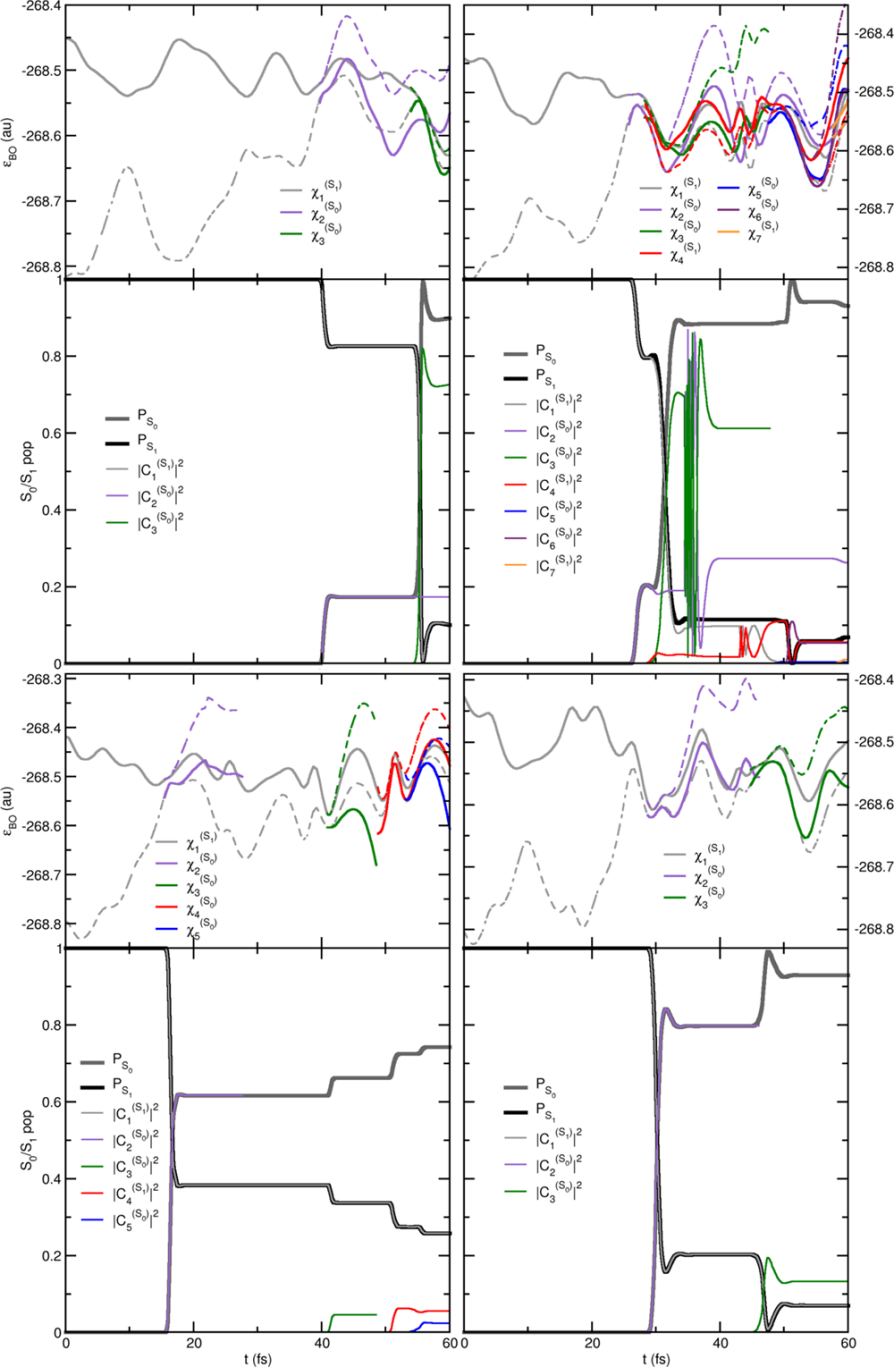
Comparing the AIMS population dynamics in ethylene for four different initial conditions (the same as those presented in Figure 2). The top panels show the electronic states of all TBFs, where the bold line shows the electronic energy of the BO state in which the TBF evolves, while the dashed lines indicate the electronic energy of the other electronic state. The bottom plots show the evolution of the population of the states (*P*_S_0__(*t*) and *P*_S_1__(*t*) as defined in eq 7, shown with thick, black, and gray lines) as well as the evolution of the squared modulus of each TBF amplitude (|*C*_*J*_^(*k*)^(*t*)|^2^).

An AIMS calculation starts with one parent TBF, assigned to a selected electronic state and with a given set of initial conditions for the nuclear positions and momenta. One can follow the electronic energy of the driving state along the dynamics of the parent TBF. This is given by a plain gray line, noted as  in Figure 9 (*J* = 1 as it is the first TBF and *k* = S_1_). The dashed line with the same color represents the electronic energy for S_0_ along the TBF evolving on S_1_. When the TBF reaches a region of strong nonadiabaticity, a new TBF is spawned onto the coupled state, here S_0_, and evolves with nuclear forces given by the electronic ground state (noted as  in Figure 9). In other words, the second TBF will have its own dynamics in S_0_ and deviate from that of the parent TBF—compare the dashed gray line (S_0_ energies on the support of ) with the plain purple line (S_0_ energies on the support of ). We stress here that the parent TBF  still exists and carries on its dynamics on S_1_, as seen from the plain gray curve. The spawning process will be repeated every time a TBF reaches a region of strong nonadiabaticity, increasing the number of TBFs (*N*_T_^*k*^(*t*)) to describe the nuclear wave function in S_0_ and S_1_.

The previous paragraph described how the TBFs evolve on the different PESs, in other words, how the moving adaptive grid spreads over time. We now need to discuss how the TDSE is solved on the support of these TBFs. This is achieved by solving the TDSE in the basis of the TBFs, leading to coupled equations of motion for the complex coefficients *C*_*J*_^(*k*)^(*t*). At the beginning of the dynamics, the parent TBF  is assigned a complex coefficient . Following a spawn, the newly created TBF  carries initially a complex coefficient  (*t*_entry_ is the time when the parent TBF originally triggered the spawning mode, when the nonadiabatic couplings crossed a certain predefined threshold). The coefficients are coupled via the TDSE and can exchange nuclear amplitude, as observed in Figure 9. We note that the population of a given electronic state is not equal to the summation of the population on each TBF evolving on this state due to the nonorthogonality of the multidimensional Gaussians. Instead, one can get the actual AIMS population in state S_0_, *P*_S_0_,_ by calculating the expectation value of the projector  using the AIMS molecular wave function
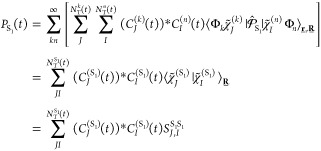
7In eq 7,  is an overlap matrix element between TBFs *J* and *I*. The AIMS populations are given by thick lines in Figure 9.

Figure 9 also highlights the conceptual difference between AIMS and SH. As every newly created TBF evolves independently, decoherence is naturally accounted for. In addition, AIMS assures at the individual trajectory level a much smoother population transfer as it does not rely on instantaneous hops but merely on Gaussians that will interact and have the possibility to transfer population between each other continuously. Indeed, all initial conditions show a stepwise deactivation process in AIMS, where multiple spawns are required. Interestingly, in one of the cases (top right plots of Figure 9), a small repopulation of the S_1_ state can be observed, mediated by back spawns to that state. In contrast, such effects are not reproduced in the corresponding SH trajectories as these are just minor population transfers that only a sufficiently large swarm of SH trajectories would capture.
